# FEASIBILITY AND ACCEPTABILITY OF A WEB-BASED COGNITIVE TRAINING PLATFORM FOR OLDER ADULTS: THE BREAKFAST TASK

**DOI:** 10.1093/geroni/igac059.2232

**Published:** 2022-12-20

**Authors:** Sharon Sanz Simon, Daniel Ben-Eliezer, Maria Pondikos, Yaakov Stern, Daniel Gopher

**Affiliations:** Columbia University, New York City, New York, United States; Technion Israel Institute of Technology, Haifa, Hefa, Israel; Columbia University, New York City, New York, United States; Columbia University, New York City, New York, United States; Technion Israel Institute of Technology, Haifa, Hefa, Israel

## Abstract

**Background:**

Developing efficient cognitive training for the older population is a major public health goal due to its potential cognitive benefits. A promising cognitive training target is executive control, critical for multitasking in everyday life. The aim of this pilot study was to establish the feasibility and acceptability of the Breakfast Task in older adults, a new web-based cognitive training platform that simulates real-life multitasking demands. Research Design and

**Methods:**

A community-based sample of 24 cognitively healthy participants aged between 60 and 75 (M = 69.12, SD = 3.83) underwent an online 5-session training protocol. Each session lasted 40 minutes and occurred twice a week at participant’s homes. Game performance was recorded, and participants completed questionnaires at baseline and after the intervention.

**Results:**

Feasibility metrics showed overall high recruitment (82.7%), adherence and retention rates (100%). Acceptability was considered good based on participant`s quantitative and qualitative responses. On average, participants rated the game as interesting, enjoyable and did not report difficulties in accessing the game online or in understanding the instructions. Moreover, participants showed a learning curve across sessions, improvement in most game outcomes and benefits from the emphasis change approach. Discussion and Implications: The findings provide preliminary support for the feasibility and acceptability of the Breakfast Task training platform with community-dwelling older adults and demonstrate potential cognitive benefits. Results suggest the value of further research investigating the Breakfast Task features and dose-response relationship, as well as its efficacy in older adults via larger randomized controlled trials.

